# The maxillary lateral incisor in the rehabilitation of cleft lip and palate

**DOI:** 10.1590/1678-7757-2017-0125

**Published:** 2018-04-18

**Authors:** Guida Paola Genovez Tereza, Marcos Antônio Corrêa dos Santos, Vivian Patricia Saldias Vargas Winckler, Ana Lúcia Pompeia Fraga de Almeida, Gisele da Silva Dalben

**Affiliations:** 1 Universidade de São Paulo, Hospital de Reabilitação de Anomalias Craniofaciais, Bauru, São Paulo, Brasil; 2 Universidade de São Paulo, Faculdade de Odontologia de Bauru, Departamento de Prótese; Hospital de Reabilitação de Anomalias Craniofaciais, Bauru, São Paulo, Brasil

**Keywords:** Incisor, Cleft lip, Cleft palate, Oral rehabilitation

## Abstract

**Objective:**

This study analyzed the maintenance of lateral incisors in the dental rehabilitation of individuals with cleft lip and palate.

**Material and Methods:**

The study was conducted on a tertiary craniofacial center and comprised retrospective analysis of panoramic and periapical radiographs of Caucasoid individuals with non-syndromic complete unilateral cleft lip and palate, analyzing all radiographs available on the records of each individual, from the first to the last up to 12 years of age. Overall, 2,826 records were reviewed to achieve a sample of 1,000 individuals. Among these, 487 individuals presented the permanent lateral incisors on both cleft and non-cleft sides, which were included in this study.

**Results:**

The results were evaluated in percentages and by descriptive statistics. The association between maintenance of the lateral incisor and timing of alveolar bone graft were analyzed by the t test. Among the 487 individuals, 265 had not completed treatment, 62 presented insufficient information, and 44 concluded the treatment elsewhere. Among the remaining 116 individuals, the lateral incisor was extracted from 88 (75.86%) of them on the cleft side (CS) and from 23 (19.83%) people on the non-cleft side (NCS). The age at accomplishment of alveolar bone graft was significantly associated with maintenance of the lateral incisor on the cleft side (p<0.01). Most extractions were indicated because of the inadequate positioning on the CS and for midline correction on the NCS. Rehabilitation was primarily completed by orthodontic movement (53 individuals on the CS and 13 individuals on the NCS).

**Conclusion:**

In conclusion, the lateral incisor on the cleft side was not maintained in most individuals. Positive relationship was observed between extraction of the lateral incisor and age at accomplishment of the alveolar bone graft, suggesting the need to anticipate the initial radiographic evaluation to enhance its maintenance and reduce the procedures required for rehabilitation.

## Introduction

Individuals with cleft lip and palate present complex skeletal deformities and are subjected to a treatment load that requires several procedures, which begin in childhood and continue up to adulthood, aiming to restore the normal morphology and function.

The treatment of alveolar defects usually requires alveolar bone graft^21^. Even though the alveolar bone graft is widely accepted by professionals for cleft treatment, there is still no consensus concerning the technique, timing and donor site^13^. This procedure was used in the 1960s in an early and primary manner, aiming to stabilize the premaxilla, allow tooth eruption in the cleft area and increase the alar base^18^. Unfortunately, the long-term follow-up revealed severe interferences in maxillary growth and frequent need for procedures of secondary alveolar bone graft^10^ ^,^ ^15^. Thus, the secondary bone graft was introduced into alveolar defects of individuals in the mixed dentition stage before eruption of the permanent canine, aiming to minimize late complications^1^. However, recently, it was demonstrated that earlier accomplishment of bone graft, in the deciduous or early mixed dentition, might support the eruption of the lateral incisor^8^ ^,^ ^14^ ^,^ ^19^. The results of studies on early bone graft demonstrated favorable graft healing without interference in maxillary growth^8^ ^,^ ^11^ ^,^ ^19^; additionally, when the graft is performed to facilitate the eruption of the lateral incisor, the cleft space may be orthodontically repaired in 100% of individuals^8^.

Despite the high success rates reported in the literature for the secondary alveolar bone graft, there are controversies concerning the age of accomplishment, suggesting the need to establish a specific treatment protocol^2^ ^,^ ^9^. However, we believe no study has demonstrated the utilization of lateral incisors in the dental rehabilitation of individuals with cleft lip or palate. The lateral incisor is directly related to the rehabilitation of these individuals, and the knowledge of its impact is fundamental to develop effective treatment protocols while minimizing the burden of care. This study analyzed (1) the prevalence of extraction of maxillary lateral incisors, (2) the reasons for extraction indication, (3) the association between maintenance of the lateral incisor on the cleft side and age at accomplishment of the alveolar bone graft; and (4) the types of treatment delivered for dental rehabilitation of individuals with cleft lip and palate.

## Material and methods

This study was approved by the Institutional Review Board of HRAC/USP (protocol no. 241/2011).

The inclusion criteria were: 1) Caucasoid individuals with non-syndromic complete unilateral cleft lip and palate, 2) presence of panoramic and periapical radiographs in the individual's files, from the first radiographs obtained up to the last up to 12 years of age, in addition to thorough dental history in the records to analyze the presence or absence of the permanent lateral incisors on the cleft and non-cleft sides, 3) individuals originally presenting permanent lateral incisors on both cleft and non-cleft sides.

**Figure 1:**
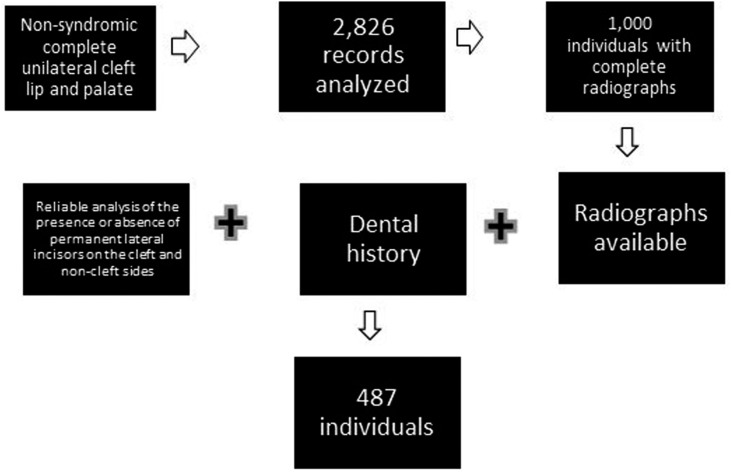
Flowchart of sample selection

A single examiner reviewed 2,826 records of individuals with non-syndromic complete unilateral cleft lip and palate, regularly registered in the institution. Among these individuals, we selected those whose records contained panoramic and periapical radiographs from the first to the last up to 12 years of age, which led to a sample of 1,000 individuals. Among these, an additional selection was performed to include only individuals whose radiographs available and dental history allowed reliable analysis of the presence or absence of permanent lateral incisors on both cleft and non-cleft sides. This led to a sample of 487 individuals who presented the permanent lateral incisors on the cleft and non-cleft sides, who were included in this study (Figure 1).

The authors did not find any study analyzing the extraction of lateral incisors for orthodontic/dental rehabilitation. For this reason, sample size calculation could not be performed. Thus, after study completion, the *post-hoc* power of the study was calculated using the following parameters: sample size 487 individuals/ percentage of extraction of lateral incisors in this sample 76%/putative percentage of extraction of lateral incisors in the overall population 1%/alpha error of 0.05, which revealed a *post-hoc* power of 100%.

This study comprised retrospective analysis of such records, searching for information about maintenance of the permanent lateral incisor on the cleft and noncleft sides for completion of dental rehabilitation. In cases with indication of lateral incisor extraction, information about the specialist who indicated the extraction and the reason for such decision was achieved, in addition to the treatment indicated for space closure in the cleft region. Secondarily, the study also evaluated the association between maintenance of the lateral incisor on the cleft side and age at accomplishment of alveolar bone graft, using the t test, at a significance level of p<0.05. The other results were evaluated in percentages and by descriptive statistics.

## Results

Among the 487 individuals previously selected who presented the permanent lateral incisor on both cleft and non-cleft sides, 265 individuals had not completed the dental rehabilitation until the onset of this study, 62 individuals exhibited insufficient information on the records, and 44 completed the treatment elsewhere. Thus, the other analyses were conducted on a sample of 116 individuals with complete unilateral cleft lip and palate, with presence of one permanent lateral incisor on both cleft and non-cleft sides, and dental rehabilitation concluded before the onset of this study.

The lateral incisor on the cleft side was maintained in 28 individuals (24.14%), while the lateral incisor on the non-cleft side was maintained in 93 individuals (80.17%).

The mean age at accomplishment of alveolar bone graft was 14.30 years (SD=4.63), ranging from 8 to 30 years. Statistically significant association was found between age at bone graft and maintenance of the lateral incisor only for the cleft side (Table 1).

**Table 1:** Maintenance of lateral incisor according to the age at accomplishment of alveolar bone graft

Maintenance of lateral incisor		Age at accomplishment of alveolar bone graft		
Cleft side	n	Mean	Standard deviation	p
Yes	28	12.25	2.43	<0.01*
No	88	14.91	4.97	
Non-cleft side	n	Mean	Standard deviation	p
Yes	93	14.19	4.48	0.73** ns
No	23	14.57	5.26	

* t test with Welch correction;

** t test

In individuals submitted to extractions, the specialist indicating them was informed only on the records of 26 individuals, with predominance of orthodontists (21), followed by maxillofacial surgeons (4) and prosthodontist (1). The reason for extraction indication was described in 16 cases (Table 2), including inadequate positioning (13), facilitation of orthodontic mechanics (1), lack of space in the dental arch (1) and lack of periodontal support (1). In cases with extraction of the lateral incisor, the corresponding space was rehabilitated by orthodontic movement with mesial movement of the canine (53), fixed prosthesis (17) and dental implant (10), without information available for the other eight individuals.

**Table 2:** Reasons for the indication of lateral incisor extraction

Reasons for indication of extraction	Cleft side	Non-cleft side
Inadequate positioning	13	3
Facilitation of orthodontic movement	1	
Lack of space in the dental arch	1	2
Lack of periodontal support	1	
Midline correction		4
No information	72	14

The specialist who indicated the extraction of the lateral incisor on the non-cleft side was described in the records of 13 individuals, all of which were indicated by the orthodontist. The reason for extraction indication was described in nine cases, including midline correction (4), inadequate positioning (3) and achievement of space at the posterior region (2). In cases of extraction of the lateral incisor, the corresponding space was rehabilitated by orthodontic movement with mesial movement of the canine (13) and fixed prosthesis (1), without information available for the other nine individuals.

## Discussion

The rehabilitation of individuals with complete unilateral cleft lip and palate is complex and involves several stages, including alveolar bone graft, which is fundamental to join the alveolar segments^1^. The main objectives of alveolar reconstruction are closure of nasal fistula, unification of maxillary segments, providing bone support for eruption of anterior teeth and for the nasal base, and allow prosthetic reconstruction including dental implants^1^ ^,^ ^3^ ^,^ ^5^.

Bone graft should be performed whenever possible to facilitate the eruption of teeth close to the cleft or orthodontic movement, either of the canine or lateral incisor^8^. Most studies consider the roots of canines to establish the timing for accomplishment of bone graft, considering one fourth and/or two thirds of its length^4^ ^,^ ^12^ ^,^ ^16^ ^,^ ^21^. However, recent studies demonstrated the possibility of alveolar bone graft in the deciduous dentition, providing sufficient bone support for eruption of central and lateral incisors with more favorable positioning of maxillary teeth^20^. Also, orthodontic space closure may be possible in 100% of cases when the bone graft is performed to facilitate the eruption of lateral incisor^7^ ^,^ ^8^. Additionally, individuals older than 12 years are four times more likely to present postoperative complications after alveolar bone graft^13^. Recently, the accomplishment of alveolar graft before eruption of the lateral incisor reduced the frequency of permanent canine impaction. This intervention does not change the risk, even though it was smaller in the group submitted to early bone graft^6^.

The eruption and adequate positioning of the permanent lateral incisor in the cleft area maintains the bone graft by mechanical stimulation, but also achieves stable functional occlusion, allowing normal maxillary growth and more harmonious facial and dental esthetics^22^.

In this study, the mean age at accomplishment of alveolar bone graft was 14.30 years (SD=4.63), ranging from 8 to 30 years. This may have been influenced by the continental dimensions of the country and predominantly low socioeconomic status of the population assisted at the institution; and this may have contributed to the low percentage of maintenance of the lateral incisor on the cleft side, as demonstrated by the statistically significant association between maintenance of the lateral incisor and age at accomplishment of alveolar bone graft (Table 1). However, this is the first study on this subject conducted at the institution and the findings should be carefully interpreted. Additionally, the presence, position and root morphology of the lateral incisor should be carefully analyzed to assure the possibility of maintenance of this tooth^18^. Thus, ideally, the maxillofacial surgeon should evaluate both the individual and the radiographs before the mixed dentition stage to properly determine the better timing for bone graft, thus allowing more favorable positioning of the lateral incisor^11^.

Our results revealed indication of 17 prostheses and 10 implants, and the space corresponding to the maxillary lateral incisor on the cleft side was closed by orthodontic mesial movement in 53 individuals (Table 3). When necessary and possible, the mesial movement of canine is favorable to reduce the utilization of prostheses and implants. Most lateral incisors on the non-cleft side were maintained. The accomplishment of bone graft before eruption of the incisors provides bone support for the eruption of these teeth, restoring the maxillary arch shape and enhancing their retention and gingival health. The burden of care may be reduced by minimizing the treatment stages and allowing earlier treatment completion, eliminating the need for rehabilitation with prostheses in adulthood^11^ ^,^ ^17^ and providing more favorable esthetic results by closing the space with a natural tooth. This aspect highlights the importance of bone graft and orthodontics in individuals with the lateral incisor.

**Table 3:** Treatment performed for dental rehabilitation

Treatment	Cleft side	Non-cleft side
Mesial movement of canine	53	13
Dental prosthesis	17	1
Dental implant	10	-
No information	8	9

Unfortunately, several records were incompletely filled by the different professionals treating the individuals in the institution. This is an inherent limitation of retrospective studies, especially those conducted in large institutions involving different specialists, such as in this case.

Based on these results, the authors concluded that 1) most of the lateral incisors (76%) adjacent to the cleft were extracted; 2) the main reason for extracting the lateral incisor was its inadequate position; 3) the earlier the bone grafting procedure is accomplished, the greater are the chances of maintaining the lateral incisor; and 4) the rehabilitation of the cleft area in most of the cases was achieved by means of orthodontic space closure.

Thus, analyzing the maintenance of lateral incisor for dental rehabilitation of individuals with complete unilateral cleft lip and palate, we observed that evaluation for alveolar bone graft is mostly performed considering the canine, i.e. when the lateral incisor is already erupted, thus missing the ideal timing of bone graft for this tooth, despite present and in good conditions. Considering the reports of success of secondary alveolar graft performed before eruption of the lateral incisor and the low rate of use of this tooth when bone graft is performed after eruption of the lateral incisor, as demonstrated in this study, the possibility of a slight anticipation in the timing of alveolar bone graft might be considered to increase the possibility of utilization of the lateral incisor. These findings suggest the need to customize the timing of orthodontic evaluation and alveolar bone graft in individuals with lateral incisor in the cleft area, considering the possibility of its eruption through the bone graft to increase the maintenance of the lateral incisor, when present, and reduce the burden of care for dental rehabilitation of individuals with cleft lip and palate.

## Conclusion

The lateral incisor on the cleft side was not maintained in most individuals. There was positive relationship between extraction of the lateral incisor and age at accomplishment of the alveolar bone graft, suggesting the need to anticipate the initial radiographic evaluation to enhance its maintenance and reduce the steps required for rehabilitation.
